# Recent trends in macromolecule-conjugated hybrid quantum dots for cancer theranostic applications

**DOI:** 10.1039/d3ra02673f

**Published:** 2023-06-20

**Authors:** Lokesh Kumar Boopathy, Thiyagarajan Gopal, Anitha Roy, Rakhee Rathnam Kalari Kandy, Madan Kumar Arumugam

**Affiliations:** a Molecular Research Laboratory, Meenakshi Medical College Hospital and Research Institute, MAHER Kanchipuram 631552 Tamil Nadu India; b Centre for Laboratory Animal Technology and Research, Sathyabama Institute of Science and Technology Chennai-600119 Tamil Nadu India; c Department of Pharmacology, Saveetha Dental College and Hospitals, Saveetha Institute of Medical and Technical Sciences Chennai-600077 Tamil Nadu India; d Marlene and Stewart Greenebaum Comprehensive Cancer Center, School of Medicine, University of Maryland Baltimore-21201 MD USA; e Cancer Biology Laboratory, Centre for Molecular and Nanomedical Sciences, Sathyabama Institute of Science and Technology Chennai-600119 Tamil Nadu India madankumarbio@gmail.com madankumar@sathyabama.ac.in +91-9942110146

## Abstract

Quantum dots (QDs) are small nanoparticles with semiconductor properties ranging from 2 to 10 nanometers comprising 10–50 atoms. The single wavelength excitation character of QDs makes it more significant, as it can excite multiple particles in a confined surface simultaneously by narrow emission. QDs are more photostable than traditional organic dyes; however, when injected into tissues, whole animals, or ionic solutions, there is a significant loss of fluorescence. HQD-based probes conjugated with cancer-specific ligands, antibodies, or peptides are used in clinical diagnosis. It is more precise and reliable than standard immunohistochemistry (IHC) at minimal protein expression levels. Advanced clinical studies use photodynamic therapy (PDT) with fluorescence imaging to effectively identify and treat cancer. Recent studies revealed that a combination of unique characteristics of QDs, including their fluorescence capacity and abnormal expression of miRNA in cancer cells, were used for the detection and monitoring progression of cancer. In this review, we have highlighted the unique properties of QDs and the theranostic behavior of various macromolecule-conjugated HQDs leading to cancer treatment.

## Abstract 2

Hybrid quantum dots can be used as a biosensor and as a cancer therapeutic material for effective cancer theranostic applications.

## Graphical abstract

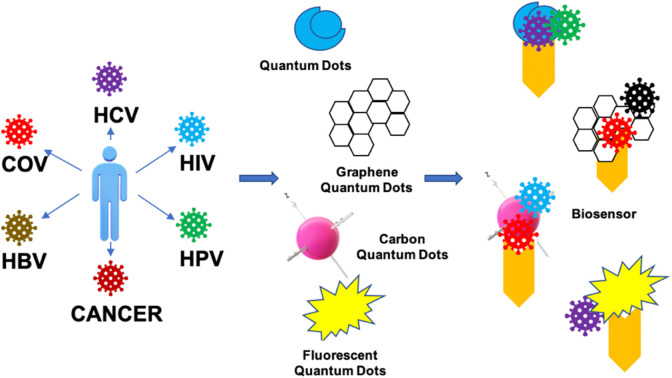

## 1. Introduction

**Fig. 1:**
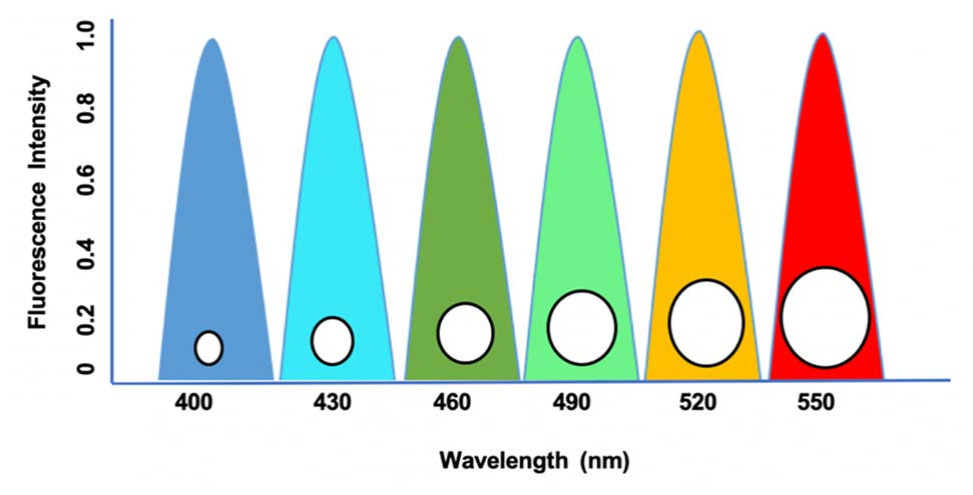
Excitation and emission profiles of QDs with symmetrical emission and narrow peaks with broad excitation at a wavelength between 400 nm to 550 nm. Intensity peak based on the absorbance and time (ns) with respect to biological samples.

Quantum dots (QDs) are small nanoparticles with semiconductor properties ranging from 2 to 10 nanometers comprising 10–50 atoms.^1^ These semiconductor-based nanoparticles are excited to higher energy levels by photons, and return to the ground state by releasing emission frequencies.^2^ These emitted frequencies regulate the movement of electrons through their ability to tune the band gaps in absorbance and emission frequencies.^3^ In connection to this, their dimension and size play a major role, as their size range of 2–6 nm is widely considered in several biological research based on their similarity with proteins and nucleic acids.^4^ The major advantage of QDs in biological research arises from their fluorophore properties at various regions that regulate the excitation spectrum, emission spectrum, photostability, and decay lifetime.^5^ The single wavelength excitation character of QDs makes it more significant as it can excite multiple particles on a confined surface simultaneously by narrow emission.^6^ Based on their narrow emission and broad absorption spectra by engineered wavelengths from the ultraviolet (UV) to infrared (IR) regions, they are applied to encode genes, proteins, and small biological molecules. Unlike other organic dyes, QDs exhibit prolonged photostability, which makes them useful in the long-term monitoring of labeled biological samples with no changes in intensity as in the case of dihydrolipoic acid (DHLA)-capped cadmium selenide–zinc sulphide (CdSe–ZnS) compared to rhodamine dye.^7^ This unique property has decreased the decay time by 30–100 ns compared to the autofluorescence background decay from other organic compounds. These characteristic features are used to determine the cancerous growths and fibroblasts in many experimental animals concerning 3T3 mouse models.^8^ QDs have superior optical properties that allow for the detection of clinically significant compounds in breast cancer diagnosis by their ability to target oligonucleotide sequences that connect with DNA or mRNA, which has been demonstrated in several studies.^9^ QD synthesis can be designed to meet various biological compounds, as its photochemical properties are influenced by the parameters of the core, shell, and coating (Fig. 1). In connection, the precise growth processes involve high annealing temperatures to produce QDs with diameters ranging from a few nanometers to a few micrometers, and the size distribution can be regulated to within 2%.^10^ In various protocols, QDs have been shown to be more photostable than traditional organic dyes. Nonetheless, when injected into tissues, whole animals, or ionic solutions, there is a significant loss of fluorescence.^11^ It has been hypothesized that this signal loss is caused by components absorbed to the surface when exposed to bodily fluids, which causes surface flaws and fluorescence quenching, or by the progressive degradation of surface ligands and coating.^12^ The commercial availability of QDs and their expanded application area due to published conjugation procedures caused an exponential rise in QD-related research articles, which have been extensively reviewed in recent years. With an emphasis on current breakthroughs for *in vitro* and *in vivo* biosensing, we summarize the developments of QD-based imaging approaches in pertinent biological materials in this review.

## 2. QDs fluorescence imaging property

**Fig. 2:**
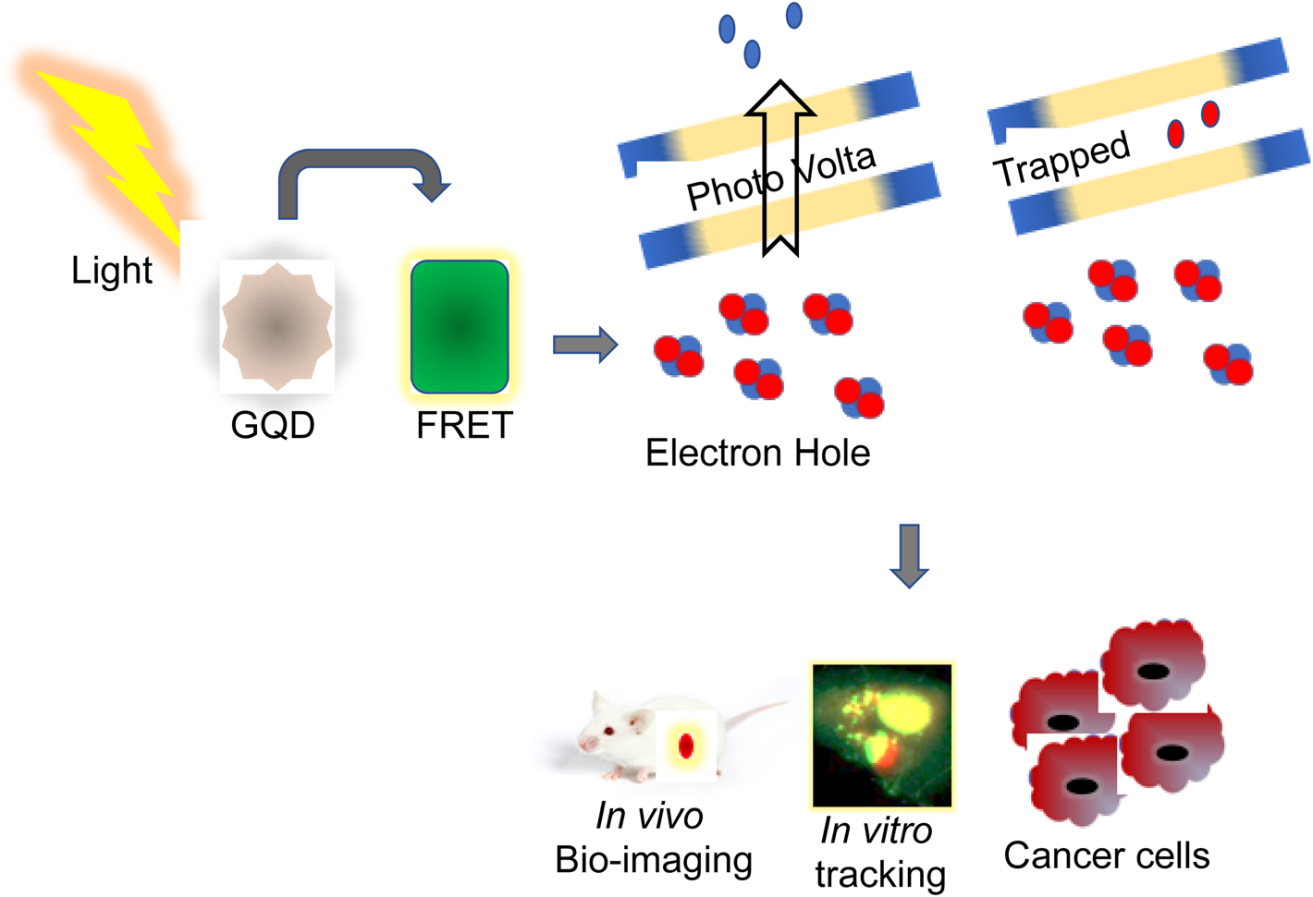
The GQDs are excited from the electron–hole through the photovoltage plates and get trapped at the UV wavelength to exhibit photoemission that can be utilized for the bioimaging of *in vivo* models, *in vitro* tracking of cancer cells, and study of apoptotic cells specifically. GQD – graphene quantum dot; FRET – fluorescence resonance energy transfer.

Recently, QDs have been extensively studied for tracing, imaging, and diagnosis of several forms of cancers. Usage of MRI is limited owing to their poor intracellular penetration and accumulation in the tumors.^13^ QDs with photothermal, photoacoustic, and photodynamic properties are employed as a nanocarrier for bioimaging and drug delivery targeting cancers.^14^ More than any other nanomaterial, semiconductor nanocrystals have influenced bioimaging research with their relationship between the size and band-gap of the semiconductor materials and their diversity in QD-based applications.^15^ Their distinct photophysical characteristics were used for animal imaging, cellular effectors, small organic molecules, biological macromolecule sensing, bioconjugation, and cell labeling.^16^ This photophysical feature of QDs is primarily responsible for their widespread use in biosensing, as the exciton (which consists of an electron–hole pair) is produced due to the absorption of photons with energy greater than the band gap. In that juncture, the chance of absorption increases with increasing excitation energy (shorter wavelength), resulting in a vast absorption spectrum and, if necessary, substantial effective Stokes shifts.^17^ This causes the intermittent fluorescence (blinking) of QDs, and non-radiative deexcitation is disrupted on the QD surface. QDs cover a broad spectrum range from the ultraviolet (UV) to the infrared (IR) by tuning the color and manipulating their size with various materials with a narrow full-width-at-half-maximum of around 20 to 30 nm and a negligible tendency of photobleaching.^18^ Utilizing the entire fluorescence property, it provides an almost infinite variety of fluorescent probes for biosensing. It is a predominant choice because each class of fluorophore has benefits such as tunable absorption and emission spectra, spectrally broad and strong absorption, small emission bands, and good photostability.^19^ QDs that have been successfully delivered can be excellent fluorophores for analyzing cellular functions and architecture. One illustration is the determination of cell motility through the phagokinetic absorption of QDs and the correlation of the cells' motion to their potential for metastasis.^20^ QDs are bound to specific targets like monitoring stem cells to look at their process during and after differentiation and the behavioral activity of the offspring. Due to their low toxicity, excellent biocompatibility, and solid photoluminescence, the QDs are used for both *in vitro* and *in vivo* bioimaging (PL).^21^ Recent studies revealed that a combination of unique characteristics of QDs, including their fluorescence capacity and abnormal expression of miRNA in cancer cells, were used for the detection and monitoring progression of cancer.^22^ Jabeen *et al.* produced highly fluorescent nitrogen-doped graphene QDs (N-GQDs) by hydrothermal method. N-GQDs were demonstrated to detect DNA damage induced by UV radiation and were reported to be used in assessing aging, skin cancer, and cell death^23^ (Fig. 2). In that connection, a nanocapsule was developed by combining highly fluorescent QDs conjugated with gelatin/chondroitin through layer-by-layer assembly. After internalization into cancer cells, the nanocarriers displayed strong fluorescence to enable tracing and imaging of the proliferation of cancer cells.^24^

Similarly, lactoferrin was coupled with the QDs nanocapsule, wherein highly fluorescent mercaptopropionic acid-capped cadmium telluride QDs were attached to lactoferrin.^25^ The nanocapsule showed an enhanced intercellular uptake with potential fluorescence activity, signifying their use in the tracing and bioimaging of cancer progression.^26^ A steady multifaceted complex of 2-dimensional nano-graphene oxide (GO) and 0-dimensional graphene quantum dots (GQD) was developed by electrostatic layer-by-layer assembly through a polyethylene imine bridge (GO-PEI-GQDs).^27^ Furthermore, mono- and complex-equivalents of the GO-PEI-GQDs complex were investigated for their bioimaging, photothermal, and oxidative stress properties in breast cancer cells, such as MDA-MB-231.^28^ These nanoparticles were used for cell imaging and as a photothermal-mediated anti-cancer therapeutic tool. In addition, Kwon *et al.* developed multiphoton cancer imaging using PEG coupled with FeSe QDs. These QDs, when attached to human epidermal growth factor receptor 2 (HER2) antibodies, displayed two-photon imaging in HER2-overexpressed MCF7 cancer cells and xenograft breast tumors in mice.^29^ The multifunctional studies demonstrated by these fluorescence HQDs could be helpful in imaging and targeted cancer therapy.^30^

**Fig. 3:**
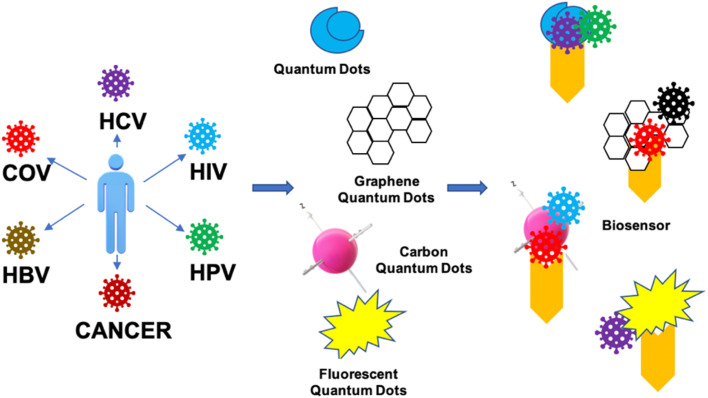
The applications of GDs by serving as a biosensor in the detection of various disease conditions by its specific attachment, which targets the desired antibodies linked with conjugated quantum materials. COV – covid; HCV – hepatitis C virus; HIV – human immunodeficiency virus; HPV – human papillomavirus; HBV – hepatitis B virus.

ODs have been successfully used to decipher biological function at the molecular level, but their potential for biosensing applications remains largely unexplored. Based on the previous reports, it is clear that QDs efficiently address some of the issues with traditional fluorophores that are currently the foundation for biosensors and bioanalytical assays due to their distinctive spectral features and physicochemical stability (Fig. 3). Sapsford *et al.* reported the advances in adapting QDs for *in vitro* biosensing applications in various immunoassays and nucleic acid detection assays.^31^ The basic characteristic of the QDs is to bind to specific biomolecules, such as oligonucleotides, proteins (peptides, antibodies or enzymes), and polymers.^32^ Chen *et al.* investigated the quick and sensitive detection of avian influenza virus in the H5N1 subtype by using QDs-based fluoroimmunoassay techniques.^33^

## 3. Role of QDs in cancer treatment

The creation of biomarkers in cell biology makes it possible to follow specific cells, including cancer cells, by combining QDs with biotinylated annexin V. This enables the functionalized QDs to attach to phosphatidylserine (PS) moieties found on the membrane of apoptotic cells, but not on healthy or necrotic cells.^34^ This helps monitor apoptotic cells and image them specifically during cancer treatment by its fundamental photostable property. In connection with that, Cao *et al.* labeled the squamous cell carcinoma cell line U14 (U14/QD800) using near-IR QDs with an emission wavelength of 800 nm (QD800).^35^ In a similar way, QDs combined with cancer antibodies have been exploited for pancreatic cancer cells to target the nucleus of living cells for fluorescent imaging.^36^ Specific quantities of QDs collected in the tumor could produce fluorescence signals that could be seen. It was indicated that QD800-based imaging, as opposed to CT and MRI, could effectively boost the sensitivity of early cancer cell diagnosis.^37^ Imaging of moving lymph nodes using QDs with various emission spectra in mouse models has been established with specific fluorescence color by simultaneously injecting five QDs into multiple places in the middle of the phalanges, the upper extremity, the ears, and the chin to observe various emission spectra of fluorescence lymphangiography.^38^ In a study, QD particles within living mice were evaluated for their mode of action into different pathways labeled with antibodies that could lead to insights into the metabolic pathways in cancerous conditions.^39^ Understanding antibody delivery mechanisms can enhance the therapeutic effectiveness in cancer treatment at that juncture. It is crucial to study the membrane interactions that are important in metastasis to better comprehend the movement of cancer cells.^40^ Using QDs tagged with antibodies against a component encouraging metastasis, Gonda *et al.* examined the membrane dynamics in metastatic cancer cells. For cancer to metastasize, changes in the membrane shape and protein dynamics dependent on its fluidity are essential.^41^ In another study, different amounts of QD-labeled cells were injected into the dorsum, back muscle, and behind the oral mucosa of naked mice. The findings showed that a significant signal requires a minimum of 104 QD-labeled cells. Over a 16 day period, the most considerable cell amount consumed (106 cells) was visible.^42^

**Fig. 4:**
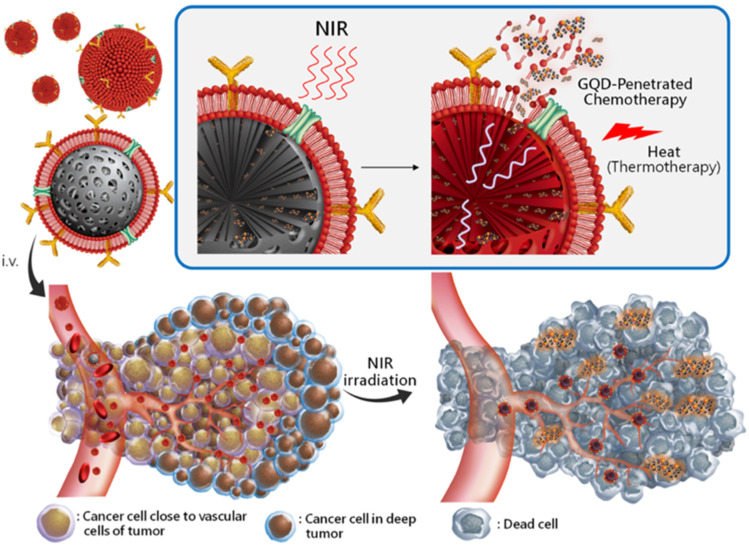
Schematic representation of targeted RBC-membrane enveloped nanosponge-mediated tumor accumulation and penetration of drug/GQDs. (i) The protein/RBCm-capped carbon/silica nanosponge delivery of DTX/GQD. (ii) The particles accumulated at the targeted sites by long-ranged motion protein/RBCm-mediated targeting. (iii) While applying NIR irradiation, the induced thermal energy leads the GQDs to penetrate and release DTX into the tumors. Reproduced with permission from ref. 47. Copyright American Chemical Society, 2019.

QD labeling to track natural killer cells was used in immunotherapeutic cell-based cancer therapy. Qdot 705 QDs were linked to the killer cells using antibodies, and a therapeutic effect was observed compared to that of unlabeled killer cells.^43^ The conjugates were intratumorally injected, and NIR fluorescence was used to capture the images. Immunotherapeutic cells marked with QDs can provide a flexible platform for precisely tracking injected therapeutic cells when used in cell-based cancer therapy. Conjugated QD offers an efficient solution with increased intensity. In the case of active targeting with cRGD-QD conjugates to passive targeting with QDs alone, the tumor PL intensity increased by almost five times.^44^ When tumor cells undergo angiogenesis, receptors like integrins are strongly expressed, and targeting such receptors for diagnostic purposes can shed light on the nature and severity of cancer.^45^ The conjugation of anti-vascular endothelial growth factor receptor 2 (VEGFR2) antibodies to QDs demonstrated the potential of QD-antibody conjugates as a diagnostic imaging tool for cancer angiogenesis.^46^ Sung *et al.* reported that a RBC membrane-enveloped nanosponge mediated tumor accumulation and QD penetration of QDs with drug-loaded graphene, which effectively reached the cancer cells and released the loaded drug DTX efficiently to kill the cancer cells (Fig. 4). Furthermore, these QDs accumulated cells treated for NIR radiation and induced thermal energy led to the GQDs penetration and release of DTX into tumors.^47^

## 4. Advantages of hybrid quantum dots (HQDs)

HQDs have received much attention in research for their potential theranostic use in various malignancies. However, QD toxicity issues resulting from their origin (ROS generation and high surface responses) and composition (heavy or inorganic materials) generated doubts about their functionalization for biological purposes.^48^ Thus, methods have been developed to reduce their toxicity and increase their biocompatibility through hybridization or functionalization with other moieties (such as polymers, lipids, polysaccharides, proteins, *etc.*), providing effective accumulation in cancerous tissue, along with avoiding their accumulation in healthy tissues. Using aqueous detergent shells with chemical groups like COOH, NH_2_, or SH, the biological molecules are linked to QDs. Several techniques, like adsorption, covalent bonding, electrostatic contact, *etc.*, have been used to form attachments.^49^ HQDs are designed as a nanocarrier to deliver drugs to the target sites, and monitor the growth and prognosis of the tumor. In connection with this, they are widely used in cancer detection and imaging of the cancerous growth and treatment of therapeutic drugs for various cancer models.^50^ QDs can interact with proteins either by structural trapping or interfacial tension. For instance, gemcitabine-loaded human serum albumin nanostructures have successfully had graphene QDs attached to their surfaces.^51^ The biocompatibility of QDs has been shown to be enhanced by embedding CdSe QDs in gelatin, as demonstrated by the fact that QD-gelatin exhibited no harmful effects on cells up to a level of 5 mg ml^−1^, while preserving their powerful luminous capabilities.^52^ In a different work, spray-dried single bovine serum albumin (BSA) nanospheres were used to physically encapsulate multiple CdTe/CdS QDs of various sizes to create multi-fluorescent nanospheres by altering the QDs size, as it is possible to change the fluorescence of the nanospheres that included a high molar ratio.^53^ Another study created multistage QD nanocarriers by fusing silica-coated QDs to the interface of the gelatin NPs to develop 100 nm nanohybrids, among other intriguing methods to improve tumor penetration.^54^ The gelatin core extravasation into the tumor tissue was followed by the hydrolysis of the ultra-small 10 nm QDs released by the increased matrix metalloproteinases in the tumor microenvironment, enabling the effective penetration into the tumor parenchyma.^55^ In terms of extending QD, systemic circulation boosts their physical stability, improves their capacity to target tumors, and lessens their toxicity.^56^

### 4.1. Theranostic behavior of HQDs in cancer treatment

HQDs-based imaging has become one of the most promising technologies for early cancer diagnosis, and demonstrates excellent performance in biomedical imaging.^57^ HQDs-based probes conjugated with cancer-specific ligands, antibodies, or peptides are used in clinical diagnosis. It is more precise and reliable than standard immunohistochemistry (IHC) at the minimal protein expression levels.^58^ Cancer therapeutic property can be achieved by quantitative detection, which can provide considerably more information for individualized treatment.^59^ HQDs have relatively large surface areas. They can be conjugated with multiple targeting ligands, like malignant cells and monoclonal antibodies, that effectively deliver the nanocarriers to the tumors and involve therapy selection.^60^ The HQDs technique will make it possible to measure numerous biomarkers simultaneously, which could produce a significant targeted ligand.^61^ These characteristics are also ideal for examining the physiological characterization of cancer cells and the tumor microenvironment, an essential topic in understanding the mechanisms behind cancer progression and creating more specialized therapy strategies.^62^

In experimental animal models, HQDs exhibit significant promise for treating tumors with a small-molecule anticancer drug Dox. Fluorescence resonance energy transfer was used in this system to quench the fluorescence of HQDs by Dox and the fluorescence of Dox by the double-stranded RNA aptamers.^63^ As a result, Dox was gradually released, enhancing the local anticancer effects. At the same time, the HQDs fluorescence served as a tool for monitoring the drug release and a versatile nanoscale scaffold for creating multifunctional nanoparticles for siRNA administration and imaging.^64^ Compared to conventional siRNA delivery agents, it can increase gene silencing activity by 10 to 20 factors and decrease cytotoxicity by 5 to 6 folds. Moreover, HQDs naturally function as double imaging probes, enabling transfection-related real-time tracking and anatomical localization. For the effective identification and treatment of cancer, advanced clinical studies use photodynamic therapy (PDT) with fluorescence imaging.^65^ These treatments give selective therapy, while leaving the immune system and normal cells unharmed, in contrast to chemotherapy and radiation therapy.^66^ The most advantageous feature of PDT is HQDs, which have efficient photostability to destroy and suppress the tumor, while using a single dose of radiation. The theranostic technique in cancer treatment may use the simultaneous delivery of chemotherapeutics and photolytic chemicals for deep tumor penetration.^67^ Both *in vitro* and *in vivo* studies have been conducted on the HQDs for cancer therapy. The most effective non-invasive cancer treatment modality with little adverse effects is PDT.^68^ It can be applied alone or in conjunction with ionizing radiation, chemotherapy, or surgery to eradicate malignant cells that were missed during resection. PDT uses photosensitizing chemicals, which are pharmacologically inert until a specific light wavelength irradiates them in the presence of oxygen, causing reactive oxygen species to be produced, and inducing tissue necrosis and cell death.^69^ Real-time monitoring of therapeutic progress is possible as hybrid QDs could be multi-modeled to treat various cancers. Combining these QDs with different nanoparticulate systems (including NPs of polymeric, lipid, and inorganic origin) to create a theranostic system for treating cancer, specifically to enhance the effectiveness of treatment for cancer.^70^

### 4.2. HQDs for anticancer drug delivery

Although cancer therapy is considered a clinically powerful tool for the treatment of cancer, it is now time to develop and synthesize a novel targeted anticancer drug delivery system to overwhelm the therapeutic problems possessed by QDs, including limited use of the drug, poor penetration, retention in tumors, undesired adverse effects to normal cells and drug resistance.^71,72^ Therefore, improving the effectiveness of chemotherapy requires sophisticated tools that allow the identification of more specific, effective, non-toxic cancer-targeting agents with potential drug release systems for improving cancer care. Chen *et al.* synthesized zinc oxide HQDs with a carbohydrate copolymer by combining two kinds of core–shell structured multifunctional nanocarriers (NCs) of ZnO QDs-tagged with Au nanoparticles as a core and an amphiphilic hyperbranched block copolymer as a shell for the cancer-targeted drug delivery system.^73^ In their synthesis, the amphiphilic hyperbranched block copolymer used as a shell had a poly(l-lactic acid) inner arm and folic acid attached with a sulfated polysaccharide isolated from the outer arm of the vine *Gynostemma pentaphyllum* Makino (GPPS-FA). Camptothecin (CPT), a pentacyclic alkaloid used as targeted anticancer drug delivery and co-loaded NCs, has been shown to enhance cytotoxicity with the rapid release of the drug in HeLa cells.^73^ The reason for this tremendous potential anticancer activity was found to be an increased cell uptake facilitated by a folate moiety attached to the NCs. A nanocomposite, GQD-ConA@Fe_3_O_4_, was developed as a nanocarrier for the loading and delivery of doxorubicin (DOX) through conjugation of GQDs and magnetic iron oxide (Fe_3_O_4_) with concanavalin A, a lectin protein. Upon being coated on a platinum electrode, the nanocomposites were observed to efficiently detect and increase the DOX susceptibility to HeLa cells.^74^ In addition, a nanoprobe was produced by conjugating carboxyl-terminated GQD with Fe_3_O_4_@SiO_2_ and functionalized with folic acid (FA), and DOX was co-loaded for intracellular drug release using the FRET mechanism. Fe_3_O_4_@SiO_2_@GQD-FA/DOX exhibited enhanced therapeutic efficacy in the HeLa cells.^75^

### 4.3. HQDs for photothermal cancer therapy

Chu *et al.* investigated red/brown and brown/black CdTe and CdSe QDs for their effect against cancer photothermal therapy. After laser irradiation with 671 nm, CdTe (710) QDs conjugated with SiO_2_ shell generated more heat and displayed significant inhibitory activity against growing mouse melanoma tumors, suggesting the photothermal potential of CdTe could be helpful in the therapeutic application of skin cancers.^76^ Fe_3_O_4_ QDs and Fe_3_O_4_–Ag_2_O QDs/cellulose fiber nanocomposites were prepared and investigated as a nanocarrier for targeted melanoma cancer therapy. The nanocomposites conjugated with the anticancer drug, including etoposide and methotrexate, exhibited outstanding drug-releasing capacity by kinetic studies, and displayed effective cytotoxic and antioxidant activity in the human malignant melanoma cancer cell line (SKMEL-3).^77^ Yang *et al.* developed carbon dots (CDs) functionalized with a nuclear localization signal peptide (NLS). NLS-CDs were transiently used to transport the anticancer drug DOX into cancer cells. DOX conjugated to NLS-CDs (DOX-CDs) *via* an acid-labile hydrazone bond showed significant induction of apoptosis in A549 cells (human lung adenocarcinoma cells). DOX-CDs inhibited tumor proliferation in A549 xenograft nude mice.^78^ Thus, these HQD complexes with enhanced therapeutic efficacy might be helpful for targeted delivery against different types of cancers.

Integrins, a cell surface receptor, are upregulated in proliferating cancer cells and are found as potential agents for targeted cancer therapy. A novel nanocarrier was synthesized using designed MiRGD peptides and GQDs. For targeted drug delivery, DOX and curcumin (Cur) were used as hydrophilic and hydrophobic drug models, respectively, and these drugs were assembled as DOX/Cur-MiRGD-GQDs peptideticles by noncovalent interactions.^79^ Administration of DOX/Cur-MiRGD-GQDs peptideticles into αv integrin overexpressed HUVEC cells or intravenous injection into 4T1-induced breast cancer mice showed an improved cellular uptake by a fluorimetric assay.^80^ Prasad *et al.* developed an HQD with superior penetration and retention of graphene QDs coupled with mesoporous silica. After near-infrared light exposure, shrinkage of solid tumors (68.75%) was observed in comparison to non-NIR light exposure. The emissive, photothermal activity and enhanced permeability made the carbon silica a promising nanohybrid agent for cancer therapy.^81^ In addition, the development of graphene QDs (GQDs)-tagged hollow copper sulfide nanoparticles (CuS NPs) by Zheng *et al.* led to the controlled intracellular drug release, and exhibited an improved photothermal-chemotherapy. DOX-CuS@GQDs NPs were synthesized by encapsulating CuS NPs with DOX, followed by the GQDs tagging on the surface of CuS NPs.^82^ Upon NIR laser irradiation, the CuS NPs readily converted light energy to heat to photothermally ablate the MDA-MB-231 cancer cells, and the photothermal ablation potential was improved by the overlaying of GQDs on the CuS NPs. These combinatorial effects (photothermal and chemotherapy) of DOX-CuS@GQDs could be used as a promising platform for synergistic therapeutics to kill cancer cells.^83^

Similarly, Li *et al.* synthesized CuInSe_2_@ZnS : Mn QDs with multifunctionality by having the potential to localize small metastases in the lungs and tumor ablation activity. After NIR laser light, the excited tumor-resident CuInSe_2_@ZnS : Mn produced thermal heat and radicals by nonradiative recombination pathways, which, in turn, ablated cancer cells and induced an anticancer immune defense to protect from the progression of tumor growth in 4T1 tumor-bearing mice.^84^ Nanocapsules were developed by combining highly-fluorescent QDs tagged with gelatin/chondroitin through layer-by-layer assembly. Drugs including celecoxib (COX-2 inhibitor) and rapamycin (mTOR inhibitor) were co-loaded into the nanocapsules to inhibit the proliferation of MCF-7 and MDA-MB-231 breast cancer cells, signifying the enhanced cytotoxicity of the nanocapsules against breast cancer cells. These hybridized nanocapsules displayed superior antitumor efficacy and nonimmunogenic response *in vivo*.^85^ The same group developed lactoferrin coupled with the QDs nanocapsule, wherein highly fluorescent mercaptopropionic acid-capped cadmium telluride QDs were attached to lactoferrin. Celecoxib, a COX-2 inhibitor, and honokiol, a lignan isolated from herbal *Magnolia grandiflora* seeds, were encapsulated in the nanocapsule, and showed an enhanced intercellular uptake with increased antitumor activity against breast cancers *in vitro* and *in vivo*.^86^

### 4.4. HQDs therapy for liver cancer

**Fig. 5:**
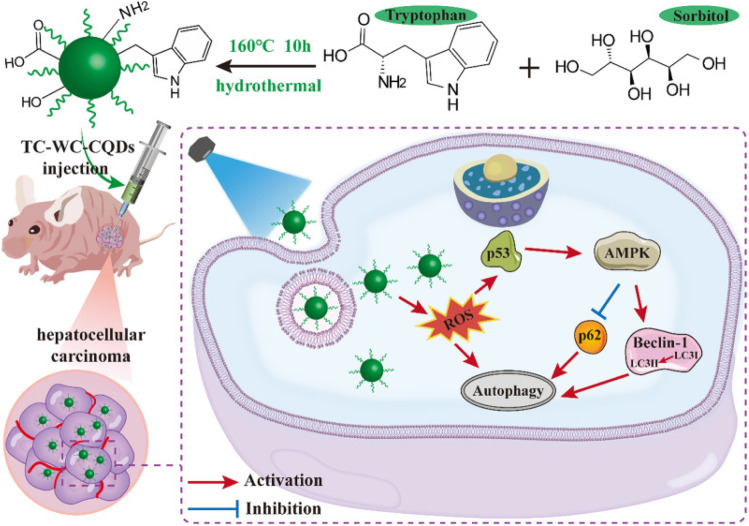
Schematic representation of the preparation of TC-WS-CQDs and light-induced antitumor mechanism of the TC-WS-CQDs, reproduced with permission from ref. 87. Copyright BMC Springer Nature, 2022.

**Fig. 6:**
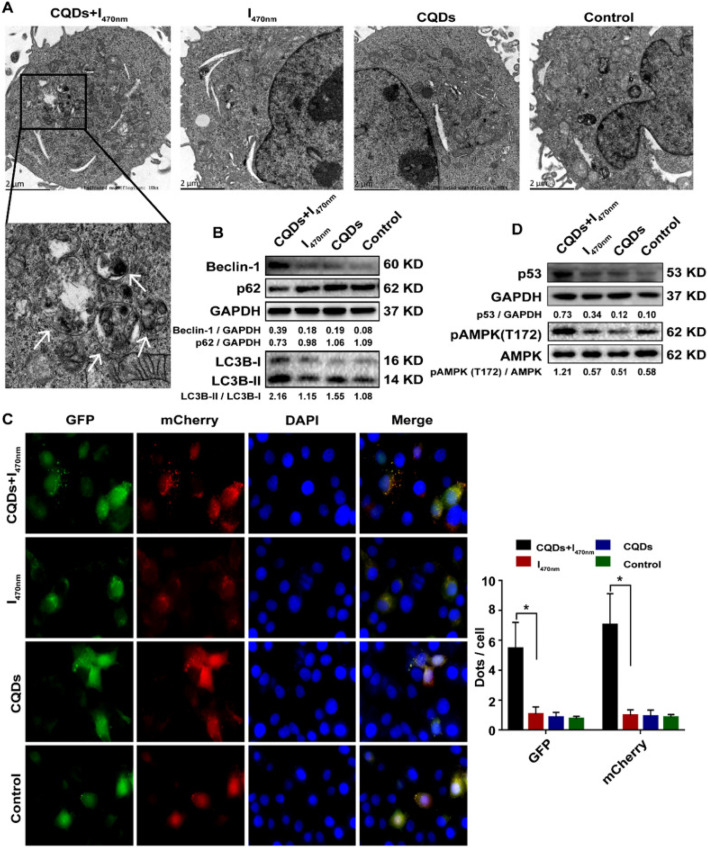
Schematic representation of the preparation of TC-WS-CQDs and their light-induced antitumor mechanism in Balb/nu mice. Induction of autophagy by photoexcited TC-WS-CQDs in Huh7 cells. (A) Detection of autophagosomes by transmission electron microscopy. Arrows indicate autophagosomes. (B) Western blot analysis of autophagy markers including LC3 conversion, expression of Beclin-1 and p62. (C) Fluorescence images of LC3 puncta expressing mcherry-GFP-LC3. Autophagosomes and autolysosomes observed in Huh7 cells. The data in (C) are mean ± SD (*n* = 3, statistical significance analyzed *via* Mann–Whitney test, *p < 0.05). (D) Western blot analysis of p53 expression and phosphorylation of AMPK. CQDs + *I*_470 nm_: TC-WS-CQDs with 470 nm irradiation, *I*_470 nm_: 470 nm irradiation without TC-WS-CQDs, CQDs: TC-WS-CQDs, control: no TC-WS-CQDs and irradiation. Reproduced with permission from ref. 87. Copyright BMC Springer Nature, 2022.

Hepatocellular carcinoma (HCC) is an end-stage liver disease with increased mortality in males compared to females.^88^ Nano-dimensional particles, including HQDs, may be alternative therapeutics with enhanced potential against patients with rapidly growing cancers. Recently, Wang *et al.* synthesized bio-friendly trichrome-tryptophan-sorbitol carbon QDs (TC-WS-CQDs) from naturally biocompatible tryptophan through the one-pot hydrothermal method,^87^ as shown in Fig. 5. Upon photoexcitation at 470 nm, TC-WS-CQDs had a potent green fluorescence emission in human hepatoma Huh7 cells. After 470 nm-irradiation, TC-WS-CQDs were observed to produce more reactive oxygen species, induction of autophagy, and inhibition of tumor proliferation through the p53-AMPK pathway in Huh7 hepatoma cells (shown in Fig. 6(A–D)), and TC-WS-CQDs were found to be non-cytotoxic in normal cells.^87^ Furthermore, the cytotoxic efficiency and mechanism of ZnO-QDs were investigated in HepG2 hepatocellular cancer cells.^89^

ZnO-QDs were shown to reduce the proliferation of HepG2 cells through significant upregulation of critical genes involved in apoptosis, including Bax, P53, and Caspase-3, with the concurrent downregulation of Bcl-2, an anti-apoptotic gene.^90^ Similarly, cadmium–selenium quantum dot nanomaterials (CdSe QDs) also demonstrated an enhanced cell death with modulation in the expression of apoptotic genes, such as Bcl2, β-catenin, Bax in the HepG2 cells.^91^ Together, these studies showed a mechanistic inhibitory effect of HQDs against various cancers, including breast cancers, melanoma, lung adenocarcinoma, and HCC.

## 5. Protein-conjugated HQDs in cancer treatment

**Fig. 7:**
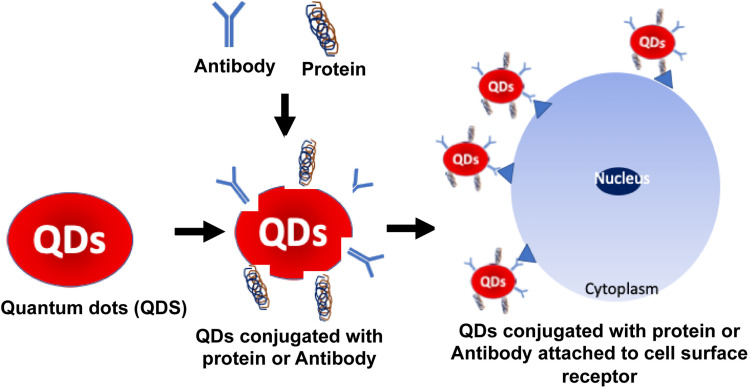
Schematic representation of the QD bioconjugate synthesis. (Step 1) QDs were activated by a linker, which can attach to the specific antibody or protein. Furthermore, these QDs conjugated antibodies or proteins were treated with cell lines for imaging or therapy. (Step 2) The antibody was specific to the cell surface receptor that can interact and attach to the cells. (Step 3) Finally, QDs were conjugated with antibodies or proteins to form the QD bioconjugate.

Protein-QDs are extensively used as nanohybrids due to their prominent role in bioimaging, as they provide high integrity in a biological system through their physical and chemical properties.^92^ The QDs used are prepared with respective metal ions that can interact with the functional groups of the amino acids, such as the carboxylic acid group, amine group, and thiol group.^93^ In connection with this, the optimum temperature is maintained to prevent protein denaturation and to achieve a high quantum yield by a narrow emission range. High photostability is the critical target in bioimaging studies on malignant tissues, and obtaining its long decay time is essential to prolong the intensity during its synthesis.^94^ Graphene QDs are conjugated with human serum albumin nanoparticles to determine the tumor cells intake capacity. Based on this data, treatment drugs are standardized over penetrating tumor cells.^95^ Many studies have mentioned the toxic characterization during the hybridization of protein QDs. To compromise that effect, encapsulation of CdSe QDs into gelatin NPs has provided positive results with biocompatibility and no toxicity in a dose-dependent manner.^96^ Cytotoxic studies were carried out with MCF-7 cell lines that expressed significant outcomes with profound biocompatibility and nontoxic with higher photostability properties.^97^ Jin *et al.* reported using QDs as fluorescent probes for receptor imaging because the QD surface is modified with biomolecules, such as antibodies, peptides, carbohydrates, and small-molecule ligands for receptors.^98^ Thus, protein-conjugated QDs are suitable for cell surface imaging, which these QDs can easily attach to the cell surface receptors based on the conjugated protein or antibodies, as shown in Fig. 7. Jin *et al.* showed the utility of ProteinA conjugated QDs, wherein HER2 (anti-human epidermal growth factor receptor 2) in KPL-4 human breast cancer cells were stained using anti-HER2 antibody conjugated Protein A-QDs.^98^ Thus, protein hybridization with QDs is essential for delivering target drugs into systemic circulation.

## 6. Carbohydrate-conjugated HQDs in cancer treatment

Polysaccharide QDs are widely used due to their distinctiveness of multifunctional groups with self-biocompatibility and biodegradability. Polysaccharide-based nanoparticles have received considerable attention as a transporter in numerous pharmacological drugs.^99^ Due to the synergistic effects, combining nanoparticles with biological molecules has received significant attention. Recently, the physicochemical and biological effects of combining natural polysaccharides with nanomaterials have been studied for high water content and surface action, which makes it more flexible in size for various drug delivery systems.^100^ The physical synthesis of polysaccharide QDs can be a very effective method for shielding many bioactive substances, particularly hydrophobic medicines and biomacromolecules, through the mechanical stability obtained by chemical cross-linking, providing a wider range of uses and more flexibility.^101^ In connection with this, conjugated polysaccharide QDs have high porosity, low weight, and a wide surface area to improve drug bioavailability and loading capacity. A recent study has reported on applying polysaccharide nano systems in treating cardiovascular disease, comprising heparin, chitosan, and numerous other substances.^102^ These polysaccharides can act in various ways, including cell signaling and adhesion, by attaching to the proper proteins and sending a message that could affect the proteins biological processes.^103^ In other cases, the binding proteins may chemically mimic the system to run the entire process naturally. Many studies have been published on the combined use of natural polysaccharides and nanomaterials for a variety of applications. For instance, it has been discovered that the drug heparin interacts with nanoparticles comprising biodegradable and inert synthetic polymers, which might open new opportunities for biosensors, tissue healing, anticancer and antitumor therapy, and improved anticoagulant efficacy.^104^

## 7. Lipid-conjugated HQDs in cancer treatment

The novel and fascinating class of nano-range delivery devices produced by the hybridization of QDs and liposomes enables the development of multifunctional drug delivery systems.^105^ These devices combine the distinctive optical characteristics of QDs with a lipid functional moiety so that hydrophilic pharmaceutical chemicals can be kept inside the internal liposome aqueous core and hydrophobic QDs inserted into the lipid bilayer.^106^ Hydrophobic QDs might be easily incorporated into the lipid bilayer when used on a thin lipid film.^107^ An increased production of LUV with some MLV, with diameters ranging from 50 nm to 50 m, was created using this technique.^108^ Compared to when hydrophobic QDs were utilized alone in toluene, the lipid bilayer's optical stability under storage and UV exposure was dramatically increased. Unlike encapsulating hydrophobic QDs, where thin film hydration is frequently used, hydrophilic QDs can be added to liposomes using several methods.^109^ To synthesize COOH-PEG-lipid coated L-QDs, thin film hydration with the hydrophobic CdSe/ZnS QDs was used. Then, sonication was used to insert the hydrophilic QDs into the aqueous core of liposomes.^110^ The single-step reverse-phase evaporation approach produced a liposomal system that transported green hydrophobic and red hydrophilic CdSe/ZnS QDs into cancer cells.^111^ After putting the QDs into the L-QDs, which led to improved photostability compared to conventional QDs, it is possible to quantitatively analyze the QDs photostability using photoluminescence (PL) spectroscopy.^112^ It is possible that the liposome-encapsulated QDs' excellent photostability will increase their effectiveness as fluorescent markers.^113^ Another study used three phospholipids with different melting temperatures to investigate the photostability of CdSe QDs encased in lipid bilayers of diverse physical states (Tm).^114^ The PL of CdSe QDs changed in a phospholipid-dependent manner while stored at room temperature. They discovered that the lipid membrane's Tm regulates the optical and chemical characteristics of the implanted QDs, and that gel-phase lipid bilayers contained the most stable QDs.^115^ The liposomal QD hybrid system significantly improves the photostability and biocompatibility of the QDs, enabling both *in vitro* and *in vivo* bio-imaging applications.^116^ The use of such hybrid liposomal devices has been discussed in a few publications. For instance, L-QDs-labeled B16F10 cells were infused into the caudal veins of C57BL/6 mice, and demonstrated that neither the host animal nor the tagged cells were harmed by the L-QD labeling.^117^

## 8. Synthetic macromolecules-conjugated HQDs in cancer treatment

Synthetic macromolecules conjugated with HQDs have photothermal and photodynamic properties that can be utilized in imaging or as a therapeutic cargo in drug administration, and are advantageous for bioimaging and treating cancer.^118^ It is susceptible and has an excellent temporal and spatial resolution, which exhibits excellent sensing and imaging cell targets.^119^ Carbon QDs (C-QDs) are easily coupled with biological species and exhibit outstanding fluorescence, photostability, and photobleaching resistance after being manufactured and distributed. In numerous studies using C-QDs with selective cell targeting, malignant cells can be specifically discovered.^120^ Y. Song *et al.* studied C-QDs conjugated with folic acid (FA) (C-dots-FA). They were able to distinguish between folate receptor (FR)-positive cancer cells and normal cells by culturing and researching NIH-3T3 and HeLa cells (FR-negative).^121^ Similarly, G. Nocito *et al.* reported that maleimide-terminated TTA1 aptamers complexed with CDs (TTA1-CDs) are primarily expressed in HeLa and C6 (a rat glioma cell line), but not in healthy CHO cells, exhibiting high fluorescence along cancer cell membranes and minimal absorption in healthy cells.^122^ Graphene QDs have been applied in various biomedical imaging in the life sciences with more efficiency. The employment of magnetic nanoparticles and GQDs in these hybrids is a potential possibility due to their distinct chemical, physical, and biological properties.^123^ A synthetic nanocomposite, GQD-ConA@Fe_3_O_4_, was studied by A. Dutta Chowdhury *et al.* for bioimaging of cancer cells *in vitro* by conjugating GQDs and magnetic iron oxide (Fe_3_O_4_) with concanavalin A, a lectin protein. This resulted in an effective approach towards cancer tracking and treatment.^124^ Upon being coated on a platinum electrode, the nanocomposites were observed to efficiently detect HeLa cells through electrochemical cyclic voltammetry techniques.^125^ Similarly, Su *et al.* developed a less cytotoxic graphene QD (GQD)@Fe_3_O_4_@SiO_2_, a luminomagnetic nanoprobe, for *in vitro* fluorescence imaging.^126^ The nanoprobe was synthesized by conjugating carboxyl-terminated GQDs with Fe_3_O_4_@SiO_2_ and functionalized with folic acid, a molecule targeting for imaging the cancer cells using the fluorescence resonance energy transfer (FRET) mechanism.^75^ In contrast, the development of near-infrared fluorescence-based carbon dots have been shown to have an effective photostability with low cytotoxicity in the H22 tumor models of the ICR mice, and reported to use this highly efficient nanomaterial for photothermal and photoacoustic imaging.^127^ The clinical importance of conjugated QDs in the diagnosis, drug administration and treatment for various disease conditions are summarized in Table 1.

**Table 1:** Depiction of the clinical importance of conjugated quantum dots in the diagnosis, drug administration and treatment for various disease conditions^a^

Types of QDs	Type of cancer	Diagnostic technique	Model	Outcome	Reference
Gelatin–CdTe QDs	Breast cancer	Confocal laser scanning microscopy	*In vitro*	Enabled synergistic growth inhibition of breast cancer cells	85
			*In vivo*		
Iron selenide (FeSe)	Breast cancer	Multiphoton microscopy	*In vitro*	*In vitro* tumour cell targeting specificity was evaluated in HER2-overexpressed MCF7 cells using FeSe QDs and *in vivo* MPM imaging was conducted in a live xenograft mouse model of human breast tumour	128
			*In vivo*		
Near-infrared QDs	Squamous carcinoma	Confocal laser scanning microscopy	*In vitro*	Detection of cell proliferation and apoptosis. *In vivo* imaging increased the sensitivity of cancer in early detection by a factor of 100 compared with traditional detection methods	35
			*In vivo*		
Cadmium–selenide and indium–gallium-phosphide	Pancreatic carcinoma breast cancer	TEM fluorescence microscopy	*In vitro*	The effect of radiofrequency field exposure after targeted nanoparticle treatment in a coculture of 2 human cancer cell lines that have differential EGFR-1 expression (a high-expressing pancreatic carcinoma, Panc-1, and a low-expressing breast carcinoma, Cama-1). Bifunctionality of fluorescent nanoparticles as agents for both cancer cell imaging and treatment	36
		Flow cytometry			
The red QD is attached to a UBI, an antimicrobial peptide. The green QD is attached to MDP, which accumulates in areas of inflammation	Charcot neuroarthropathy	Fluorescence microscopy	*In vitro*	QD-based method for distinguishing CN with sterile inflammation from osteomyelitis that does not require multiple and frequent imaging modalities	129
Aptamer (Apt-)-doxorubicin (Dox) conjugate system [QD-Apt(Dox)]	Prostate cancer	Confocal laser scanning microscopy	*In vitro*	Sterile inflammation from osteomyelitis that does not require multiple and frequent imaging modalities	130
Near-infrared luminescent	Oral squamous cell carcinoma	*In Vivo* imaging system FX pro	*In vitro*	Great promise for the early diagnosis, visual observation, and individualized treatment of oral cancer	131
			*In vivo*		
Immunohistochemical (IHC) technique and trastuzumab-conjugated Qds (IHC-Qds)	Breast cancer	Single-particle imaging system	*In vitro*	The novel IHC-QDs method could achieve autofluorescence subtracted imaging of tumour cells and rapid diagnosis of the HER2-expression level, which overcame the disadvantages of traditional IHC protocol	132
Silver–indium–sulphide QDs	Colon cancer	Fluorescence imaging	*In vitro*	Elicited significant cell death due to enhanced light-induced ROS generation and apoptotic/necrotic cell death	48
CdSe/ZnS coated with silica, polyamidoamine and PEG	Colon cancer	MRI imaging system	*In vitro*	Early tumor detection using MRI	133
Mercaptoundecanoic acid-coated CdTe/CdSe/ZnSe QD conjugated to anti-HER2 mAb	Breast cancer	Fluorescence imaging	*In vitro*	Anti-HER2 targeted breast cancer therapy	134
124I-cRGDY–PEG–C dots	Melanoma	Targeted molecular therapy and imaging	Human	Tumor visualization and targeted therapy	135
CuInS(2)/ZnS QD	Breast cancer	Sentinel lymph node tracing in mice breast cancer model	*In vivo*	Visualization of lymph nodes after 5 min, stable signal regardless of the metastatic invasion	136

a The clinical importance of conjugated QDs in the diagnosis, drug administration and treatment for various disease conditions. The experimental evidence shows the various diagnostic tools that serve as an important factor in the early diagnosis and treatment of cancer cells in both *in vivo* and *in vitro* studies.

## 9. QDs cytotoxicity and its drawbacks

Although QDs have attracted much attention and have started to be used in preclinical settings, one significant unresolved concern is their potential for cytotoxicity. According to the previous reports, QD cytotoxicity can be explained by their physicochemical characteristics, such as their size, surface charge, ligands nature, and interactions with other molecules already present in biological media.^137^ For instance, when capping QDs, mercaptoacetic acid has the potential to be cytotoxic to the cells. A deleterious effect on cells becomes evident for mercaptoacetic acid concentrations over 6 μM and for PEG-silica-coated QDs concentrations above 30 μM.^138^ Additionally, the metallic core of the QDs or the process by which the core dissolves may be poisonous if the coating is disturbed. The ions used in the core of QDs, cadmium and selenium, are known to be cytotoxic.^139^ Also, unfavorable *in vivo* results due to shell erosion are a possibility. The composition of QDs, which was seen in *in vitro* studies, suggests that QDs might be detrimental.^140^ Numerous other investigations have also revealed that QDs may be systemically distributed, and may accumulate in organs and tissues like the kidney, spleen, and liver.^138^ Lovric *et al.* reported that rat pheochromacytoma cells were found to be hazardous when exposed to CdTe QDs coated with mercaptoacetic acid.^141^ Previous studies reported that the QDs cytotoxicity appears to be dose-dependent, as 5 billion QDs micelles injected into xenopus blastomeres resulted in aberrant cell morphologies in contrast to when 2 billion micelles were injected.^140^ For instance, experiments by Jaiswal *et al.* using acute exposures of cells to QDs for 15 min to 2 h observations showed no cytotoxicity.^142^ Hanaki *et al.* used exposure times that were comparable. However, investigations with exposure periods ranging from two hours to many days typically revealed that the QDs exposure caused cytotoxicity.^143^ Hence, each QDs type will need to be evaluated individually for its possible toxicity. It is likely that grouping or categorizing QDs according to their potential toxicities only based on size or other physicochemical attributes would prove to be challenging early on.

## 10. Conclusion and future directions

HQDs are organic and biocompatible with alluring bright nanomaterial properties, which make it possible as an optical imaging platform. Through proof-of-concept experiments, recent investigations on QDs have revealed diverse physical and chemical characteristics. Despite the encouraging news regarding QDs applications, the specific mechanism of cellular absorption and the long-term toxicological effects remain unknown. The shape, physiochemical properties, surface chemistry, and formulation of QDs are just a few factors affecting their pharmacokinetics and bio-distribution. Long-wavelength excitation is required for fluorescence imaging to simultaneously increase tissue penetration and resolution. Most of the research in recent years has focused on creating novel sensing probe chemicals to improve the sensor properties, including the selectivity, sensitivity, and biocompatibility. Among the synthesized nanomaterials, HQDs are frequently used to build the sensor's surface for the interaction of molecular components in the biological system. The research field now has numerous dimensions, where the dynamics of its reaction are exposed to stimuli in depth due to the continued introduction of external stimuli. HQDs act as a unique tool in both imaging and probing, allowing a study of fundamental molecular mechanisms and chemistry at the cellular level.

## Author contributions

M. K. A. developed the idea and structure of the review article. M. K. A and L. K. B. wrote the paper using the materials supplied by T. G., A. R., and R. R. K. K, and revised and improved the manuscript. M. K. A supervised the manuscript. All the authors have given approval to the final version of the manuscript.

## Conflicts of interest

The authors declare no conflict of interest.

## Supplementary Material

## References

[cit1] Bagher A. M. (2016). Sens. Transducers.

[cit2] Chatterjee D. K., Gnanasammandhan M. K., Zhang Y. (2010). Small.

[cit3] Florescu M., Lee H., Puscasu I., Pralle M., Florescu L., Ting D. Z., Dowling J. P. (2007). Sol. Energy Mater. Sol. Cells.

[cit4] Niemeyer C. M. (2000). Curr. Opin. Chem. Biol..

[cit5] Tandale P., Choudhary N., Singh J., Sharma A., Shukla A., Sriram P., Soni U., Singla N., Barnwal R. P., Singh G. (2021). Biochem. Biophys. Rep..

[cit6] Smith A. M., Gao X., Nie S. (2004). Photochem. Photobiol..

[cit7] Jamieson T., Bakhshi R., Petrova D., Pocock R., Imani M., Seifalian A. M. (2007). Biomaterials.

[cit8] Gu Z., Yan L., Tian G., Li S., Chai Z., Zhao Y. (2013). Adv. Mater..

[cit9] Thakor A. S., Gambhir S. S. (2013). Ca-Cancer J. Clin..

[cit10] Efros A. L., Brus L. E. (2021). ACS Nano.

[cit11] Xing Y., Rao J. (2008). Cancer Biomarkers.

[cit12] Lynch I., Dawson K. A. (2008). Nano today.

[cit13] Zhang Y., Li M., Gao X., Chen Y., Liu T. (2019). J. Hematol. Oncol..

[cit14] Lim W. Q., Phua S. Z. F., Xu H. V., Sreejith S., Zhao Y. (2016). Nanoscale.

[cit15] Martynenko I., Litvin A., Purcell-Milton F., Baranov A., Fedorov A., Gun'Ko Y. (2017). J. Mater. Chem. B.

[cit16] Wegner K. D., Hildebrandt N. (2015). Chem. Soc. Rev..

[cit17] Shabbir H., Wojnicki M. (2023). Electronics.

[cit18] Gregersen S. , Doktorski rad. Københavns, Københavns Universitet, 2014

[cit19] Hildebrandt N., Spillmann C. M., Algar W. R., Pons T., Stewart M. H., Oh E., Susumu K., Diaz S. A., Delehanty J. B., Medintz I. L. (2017). Chem. Rev..

[cit20] Medintz I. L., Uyeda H. T., Goldman E. R., Mattoussi H. (2005). Nat. Mater..

[cit21] Wang J., Zhang P., Huang C., Liu G., Leung K. C.-F., Wáng Y. X. J. (2015). Langmuir.

[cit22] Ahmad J., Garg A., Mustafa G., Ahmad M. Z., Aslam M., Mishra A. (2023). Electronics.

[cit23] Tabish T. A., Scotton C. J., J Ferguson D. C., Lin L., der Veen A. v., Lowry S., Ali M., Jabeen F., Ali M., Winyard P. G. (2018). Nanomedicine.

[cit24] AbdElhamid A., Helmy M., Ebrahim S., Bahey-El-Din M., Zayed D. (2018). Nanomedicine.

[cit25] Bukhari S. I., Imam S. S., Ahmad M. Z., Vuddanda P. R., Alshehri S., Mahdi W. A., Ahmad J. (2021). Pharmaceutics.

[cit26] Ly N. H., Joo S.-W. (2020). J. Mater. Chem. B.

[cit27] Silvestri A., Criado A., Prato M. (2021). Faraday Discuss..

[cit28] Hoseini-Ghahfarokhi M., Mirkiani S., Mozaffari N., Abdolahi Sadatlu M. A., Ghasemi A., Abbaspour S., Akbarian M., Farjadian F., Karimi M. (2020). Int. J. Nanomed..

[cit29] Kwon J., Jun S., Choi S., Mao X., Kim J., Koh E., Kim Y.-H., Kim S.-K., Hwang D., Kim C.-S. (2019). Sci. Adv..

[cit30] Rahman M., Akhter S., Ahmad M. Z., Ahmad J., Addo R. T., Ahmad F. J., Pichon C. (2015). Nanomedicine.

[cit31] Sapsford K. E., Pons T., Medintz I. L., Mattoussi H. (2006). Sensors.

[cit32] Li M., Chen T., Gooding J. J., Liu J. (2019). ACS Sens..

[cit33] Chen L., Sheng Z., Zhang A., Guo X., Li J., Han H., Jin M. (2010). Luminescence.

[cit34] Zeng W., Wang X., Xu P., Liu G., Eden H. S., Chen X. (2015). Theranostics.

[cit35] Cao Y. a., Yang K., Li Z., Zhao C., Shi C., Yang J. (2010). Nanotechnology.

[cit36] Glazer E. S., Curley S. A. (2010). Cancer.

[cit37] Yousefi F., Nabipour I., Kalantarhormozi M., Assadi T., Raeisi A., Assadi M. (2015). Med. Hypotheses.

[cit38] Altavilla C. and Ciliberto E., Inorganic Nanoparticles: Synthesis, Applications, and Perspectives, CRC Press, 2017

[cit39] Ghaderi S., Ramesh B., Seifalian A. M. (2011). J. Drug Targeting.

[cit40] Shi J., Kantoff P. W., Wooster R., Farokhzad O. C. (2017). Nat. Rev. Cancer.

[cit41] Gonda K., Watanabe T. M., Ohuchi N., Higuchi H. (2010). J. Biol. Chem..

[cit42] Yang K., Cao Y. A., Shi C., Li Z. G., Zhang F. J., Yang J., Zhao C. (2010). Oral Oncol..

[cit43] Chakraborty P., Das S. S., Dey A., Chakraborty A., Bhattacharyya C., Kandimalla R., Mukherjee B., Gopalakrishnan A. V., Singh S. K., Kant S., Nand P., Ojha S., Kumar P., Jha N. K., Jha S. K., Dewanjee S. (2022). J. Controlled Release.

[cit44] Radenkovic D., Kobayashi H., Remsey-Semmelweis E., Seifalian A. M. (2016). Nanomedicine.

[cit45] Ludwig B. S., Kessler H., Kossatz S., Reuning U. (2021). Cancers.

[cit46] Liu B., Jiang B., Zheng Z., Liu T. (2019). J. Lumin..

[cit47] Sung S. Y., Su Y. L., Cheng W., Hu P. F., Chiang C. S., Chen W. T., Hu S. H. (2019). Nano Lett..

[cit48] Hashemkhani M., Loizidou M., MacRobert A. J., Yagci Acar H. (2022). Inorg. Chem..

[cit49] Kim D. C., Kang D. J. (2008). Sensors.

[cit50] Bechet D., Mordon S. R., Guillemin F., Barberi-Heyob M. A. (2014). Cancer Treat. Rev..

[cit51] Rehman M. U., Khan A., Imtiyaz Z., Ali S., Makeen H. A., Rashid S., Arafah A. (2022). Cancer Treat. Rev..

[cit52] Zhang F., Yi D., Sun H., Zhang H. (2014). J. Nanosci. Nanotechnol..

[cit53] Xue Z., Zhang Y., Yu W., Zhang J., Wang J., Wan F., Kim Y., Liu Y., Kou X. (2019). Anal. Chim. Acta.

[cit54] Elzoghby A. O., Hemasa A. L., Freag M. S. (2016). J. Controlled Release.

[cit55] Zhao W., Yu X., Peng S., Luo Y., Li J., Lu L. (2021). J. Nanobiotechnol..

[cit56] Sun T., Zhang Y. S., Pang B., Hyun D. C., Yang M., Xia Y. (2014). Angew. Chem., Int. Ed. Engl..

[cit57] Xiang Y., Hu C., Wu G., Xu S., Li Y. (2023). TrAC, Trends Anal. Chem..

[cit58] Byers R. J., Hitchman E. R. (2011). Prog. Histochem. Cytochem..

[cit59] Yallapu M. M., Othman S. F., Curtis E. T., Bauer N. A., Chauhan N., Kumar D., Jaggi M., Chauhan S. C. (2012). Int. J. Nanomed..

[cit60] Pérez-Herrero E., Fernández-Medarde A. (2015). Eur. J. Pharm. Biopharm..

[cit61] Dhas N., Pastagia M., Sharma A., Khera A., Kudarha R., Kulkarni S., Soman S., Mutalik S., Barnwal R. P., Singh G., Patel M. (2022). J. Controlled Release.

[cit62] Mukherjee A., Shim Y., Myong Song J. (2016). Biotechnol. J..

[cit63] Ghosal K., Sarkar K. (2018). ACS Biomater. Sci. Eng..

[cit64] Tavakolifard S., Biazar E. (2016). Nano Biomed. Eng..

[cit65] Ackroyd R., Kelty C., Brown N., Reed M. (2001). Photochem. Photobiol..

[cit66] De Munck J., Binks A., McNeish I. A., Aerts J. L. (2017). J. Leukoc. Biol..

[cit67] Khizar S., Alrushaid N., Alam Khan F., Zine N., Jaffrezic-Renault N., Errachid A., Elaissari A. (2023). Int. J. Pharm..

[cit68] Fusco L., Gazzi A., Peng G., Shin Y., Vranic S., Bedognetti D., Vitale F., Yilmazer A., Feng X., Fadeel B., Casiraghi C., Delogu L. G. (2020). Theranostics.

[cit69] Rai P., Mallidi S., Zheng X., Rahmanzadeh R., Mir Y., Elrington S., Khurshid A., Hasan T. (2010). Adv. Drug Deliv. Rev..

[cit70] Nam J., Won N., Bang J., Jin H., Park J., Jung S., Jung S., Park Y., Kim S. (2013). Adv. Drug Deliv. Rev..

[cit71] Debela D. T., Muzazu S. G., Heraro K. D., Ndalama M. T., Mesele B. W., Haile D. C., Kitui S. K., Manyazewal T. (2021). SAGE Open Med..

[cit72] Gavas S., Quazi S., Karpiński T. M. (2021). Nanoscale Res. Lett..

[cit73] Chen T., Zhao T., Wei D., Wei Y., Li Y., Zhang H. (2013). Carbohydr. Polym..

[cit74] Dutta Chowdhury A., Ganganboina A. B., Tsai Y. C., Chiu H. C., Doong R. A. (2018). Anal. Chim. Acta.

[cit75] Su X., Chan C., Shi J., Tsang M. K., Pan Y., Cheng C., Gerile O., Yang M. (2017). Biosens. Bioelectron..

[cit76] Chu M., Pan X., Zhang D., Wu Q., Peng J., Hai W. (2012). Biomaterials.

[cit77] Fakhri A., Tahami S., Nejad P. A. (2017). J. Photochem. Photobiol., B.

[cit78] Yang L., Wang Z., Wang J., Jiang W., Jiang X., Bai Z., He Y., Jiang J., Wang D., Yang L. (2016). Nanoscale.

[cit79] Hamidi H., Ivaska J. (2018). Nat. Rev. Cancer.

[cit80] Moasses Ghafary S., Rahimjazi E., Hamzehil H., Modarres Mousavi S. M., Nikkhah M., Hosseinkhani S. (2022). Nanomedicine.

[cit81] Prasad R., Jain N. K., Yadav A. S., Jadhav M., Radharani N. N. V., Gorain M., Kundu G. C., Conde J., Srivastava R. (2021). ACS Appl. Bio Mater..

[cit82] Zheng S., Jin Z., Han C., Li J., Xu H., Park S., Park J.-O., Choi E., Xu K. (2020). J. Mater. Sci..

[cit83] Karthikeyan L., Vivek R. (2022). Adv. Cancer Biol.: Metastasis.

[cit84] Li Y., Zhang P., Tang W., McHugh K. J., Kershaw S. V., Jiao M., Huang X., Kalytchuk S., Perkinson C. F., Yue S., Qiao Y., Zhu L., Jing L., Gao M., Han B. (2022). ACS Nano.

[cit85] AbdElhamid A. S., Helmy M. W., Ebrahim S. M., Bahey-El-Din M., Zayed D. G., Zein El Dein E. A., El-Gizawy S. A., Elzoghby A. O. (2018). Nanomedicine.

[cit86] AbdElhamid A. S., Zayed D. G., Helmy M. W., Ebrahim S. M., Bahey-El-Din M., Zein-El-Dein E. A., El-Gizawy S. A., Elzoghby A. O. (2018). Nanomedicine.

[cit87] Wang Y., Chen J., Tian J., Wang G., Luo W., Huang Z., Huang Y., Li N., Guo M., Fan X. (2022). J. Nanobiotechnol..

[cit88] Wu E. M., Wong L. L., Hernandez B. Y., Ji J. F., Jia W., Kwee S. A., Kalathil S. (2018). Hepatoma Res..

[cit89] Anjum S., Hashim M., Malik S. A., Khan M., Lorenzo J. M., Abbasi B. H., Hano C. (2021). Cancers.

[cit90] Ahmad J., Wahab R., Siddiqui M. A., Musarrat J., Al-Khedhairy A. A. (2015). Bioprocess Biosyst. Eng..

[cit91] Rahman M. M., Opo F., Asiri A. M. (2021). J. Biomed. Nanotechnol..

[cit92] Kargozar S., Hoseini S. J., Milan P. B., Hooshmand S., Kim H. W., Mozafari M. (2020). Biotechnol. J..

[cit93] Li G., Fei X., Liu H., Gao J., Nie J., Wang Y., Tian Z., He C., Wang J. L., Ji C., Oron D., Yang G. (2020). ACS Nano.

[cit94] Takahashi R., Kono K., Tarucha S., Ono K. (2011). Phys. Rev. Lett..

[cit95] Cheng Z., Li M., Dey R., Chen Y. (2021). J. Hematol. Oncol..

[cit96] Caballero-Díaz E., Guzmán-Ruiz R., Malagón M. M., Simonet B. M., Valcárcel M. (2014). J. Hazard. Mater..

[cit97] Prabhakar A. K., Ajith M. P., Ananthanarayanan A., Routh P., Mohan B. C., Thamizhchelvan A. M. (2022). OpenNano.

[cit98] Jin T., Tiwari D. K., Tanaka S., Inouye Y., Yoshizawa K., Watanabe T. M. (2010). Mol. BioSyst..

[cit99] Yubia D. A., Carvajal-Millan E., Campa-Mada A., Lizardi-Mendoza J., Rascon-Chu A., Tanori-Cordova J., Luisa Martínez-Lopez A. (2021). Polysaccharides.

[cit100] Li Y., Zheng X., Chu Q. (2021). Nano Today.

[cit101] Kurczewska J. (2022). Polymers.

[cit102] Taneja P., Sharma S., Sinha V. B., Yadav A. K. (2021). Life Sci..

[cit103] Kim S. H., Turnbull J., Guimond S. (2011). J. Endocrinol..

[cit104] Salatin S., Yari Khosroushahi A. (2017). J. Cell. Mol. Med..

[cit105] Bhatia S. (2016). Natural Polymer Drug Delivery Systems. Nanoparticles, Plants, and Algae.

[cit106] Al-Jamal W. T., Kostarelos K. (2011). Acc. Chem. Res..

[cit107] De Leo V., Maurelli A. M., Giotta L., Catucci L. (2022). Colloids Surf., B.

[cit108] Mayer L. D., Bally M. B., Hope M. J., Cullis P. R. (1986). Chem. Phys. Lipids.

[cit109] Mansur H. S. (2010). Wiley Interdiscip. Rev.: Nanomed. Nanobiotechnol..

[cit110] Zhou J., Yang Y., Zhang C. Y. (2015). Chem. Rev..

[cit111] Souza S. O., Lira R. B., Cunha C. R. A., Santos B. S., Fontes A., Pereira G. (2021). Top. Curr. Chem..

[cit112] Zheng W., Liu Y., West A., Schuler E. E., Yehl K., Dyer R. B., Kindt J. T., Salaita K. (2014). J. Am. Chem. Soc..

[cit113] Awad N. S., Haider M., Paul V., AlSawaftah N. M., Jagal J., Pasricha R., Husseini G. A. (2021). Pharmaceutics.

[cit114] Wang T., Bai J., Jiang X., Nienhaus G. U. (2012). ACS Nano.

[cit115] Malekkhaiat Häffner S., Malmsten M. (2017). Adv. Colloid Interface Sci..

[cit116] Liang Z., Khawar M. B., Liang J., Sun H. (2021). Front. Oncol..

[cit117] Ruttala H. B., Ko Y. T. (2015). Colloids Surf., B.

[cit118] Battigelli A., Ménard-Moyon C., Da Ros T., Prato M., Bianco A. (2013). Adv. Drug Deliv. Rev..

[cit119] Meng X., Pang X., Zhang K., Gong C., Yang J., Dong H., Zhang X. (2022). Small.

[cit120] Singh I., Arora R., Dhiman H., Pahwa R. (2018). Turkish J. Pharm. Sci..

[cit121] Song Y., Shi W., Chen W., Li X., Ma H. (2012). Fluorescent carbon nanodots conjugated with folic acid for distinguishing folate-receptor-positive cancer cells from normal cells. J. Mater. Chem..

[cit122] Nocito G., Calabrese G., Forte S., Petralia S., Puglisi C., Campolo M., Esposito E., Conoci S. (2021). Cancers.

[cit123] Wang Z., Hu T., Liang R., Wei M. (2020). Front. Chem..

[cit124] Dutta Chowdhury A., Ganganboina A. B., Tsai Y.-c., Chiu H.-c., Doong R.-a. (2018). Anal. Chim. Acta.

[cit125] Li Y., Fang L., Cheng P., Deng J., Jiang L., Huang H., Zheng J. (2013). Biosens. Bioelectron..

[cit126] Li K., Zhao X., Wei G., Su Z. (2018). Curr. Med. Chem..

[cit127] Bao X., Yuan Y., Chen J., Zhang B., Li D., Zhou D., Jing P., Xu G., Wang Y., Holá K., Shen D., Wu C., Song L., Liu C., Zbořil R., Qu S. (2018). Light: Sci. Appl..

[cit128] Kwon J., Jun S. W., Choi S. I., Mao X., Kim J., Koh E. K., Kim Y.-H., Kim S.-K., Hwang D. Y., Kim C.-S., Lee J. (2019). Sci. Adv..

[cit129] Zeng M., Xu Z., Song Z. Q., Li J. X., Tang Z. W., Xiao S., Wen J. (2023). World J. Orthop..

[cit130] Bagalkot V., Zhang L., Levy-Nissenbaum E., Jon S., Kantoff P. W., Langer R., Farokhzad O. C. (2007). Nano Lett..

[cit131] Yang K., Zhang F. J., Tang H., Zhao C., Cao Y. A., Lv X. Q., Chen D., Li Y. D. (2011). Int. J. Nanomed..

[cit132] Miyashita M., Gonda K., Tada H., Watanabe M., Kitamura N., Kamei T., Sasano H., Ishida T., Ohuchi N. (2016). Cancer Med..

[cit133] Bakalova R., Zhelev Z., Kokuryo D., Spasov L., Aoki I., Saga T. (2011). Int. J. Nanomed..

[cit134] Rizvi S. B., Rouhi S., Taniguchi S., Yang S. Y., Green M., Keshtgar M., Seifalian A. M. (2014). Int. J. Nanomed..

[cit135] Phillips E., Penate-Medina O., Zanzonico P. B., Carvajal R. D., Mohan P., Ye Y., Humm J., Gönen M., Kalaigian H., Schöder H., Strauss H. W., Larson S. M., Wiesner U., Bradbury M. S. (2014). Sci. Transl. Med..

[cit136] Helle M., Cassette E., Bezdetnaya L., Pons T., Leroux A., Plénat F., Guillemin F., Dubertret B., Marchal F. (2012). PLoS One.

[cit137] Oh E., Liu R., Nel A., Gemill K. B., Bilal M., Cohen Y., Medintz I. L. (2016). Nat. Nanotechnol..

[cit138] Alivisatos A. P. (1996). Science.

[cit139] Nikazar S., Sivasankarapillai V. S., Rahdar A., Gasmi S., Anumol P. S., Shanavas M. S. (2020). Biophys. Rev..

[cit140] Hardman R. (2006). Environ. Health Perspect..

[cit141] Lovric J., Bazzi H. S., Cuie Y., Fortin G. R., Winnik F. M., Maysinger D. (2005). J. Mol. Med..

[cit142] Jaiswal J. K., Mattoussi H., Mauro J. M., Simon S. M. (2003). Nat. Biotechnol..

[cit143] Hanaki K., Momo A., Oku T., Komoto A., Maenosono S., Yamaguchi Y., Yamamoto K. (2003). Biochem. Biophys. Res. Commun..

